# Combining urban scaling and polycentricity to explain socio-economic status of urban regions

**DOI:** 10.1371/journal.pone.0218022

**Published:** 2019-06-14

**Authors:** Amin Khiali-Miab, Maarten J. van Strien, Kay W. Axhausen, Adrienne Grêt-Regamey

**Affiliations:** 1 Planning of Landscape and Urban Systems, ETH Zürich, Zürich, Switzerland; 2 Institute for Transport Planning and Systems, ETH Zürich, Zürich, Switzerland; Bruno Kessler Foundation, ITALY

## Abstract

The fast pace of urbanisation may benefit or be detrimental to the socio-economic status of urban areas. Understanding how the configuration of urban areas influences the socio-economic status of their inhabitants is of crucial importance for urban planning. In theory, urban scaling laws and polycentric development are two well-known concepts developed to increase our understanding of urbanisation and its socio-economic effects. In practice, however, they fall short to explain the socio-economic status of urban regions. The urban scaling concept is constructed from a theoretical perspective, but functional relationships between urban centres are not taken into account in scaling models. In contrast, the concept of polycentricity is developed from a practical perspective and incorporates the socio-economic effect of relationships between urban centres in the process of urban development. However, polycentricity lacks a theoretical foundation, which would explain the socio-economic status of urban regions. In this study, we assess whether combining both concepts improves the ability to explain personal incomes in metropolitan areas in Switzerland. We first delineated metropolitan areas by implementing a modularity maximisation algorithm on the settlement network. Nodes in this network are Swiss municipalities and links are inter-municipal commuter flows. We found a strong relationship between the hierarchical organisation of functional connections within metropolitan areas and the socio-economic status of these areas. Both concepts were complementary and combining them proved to enhance the ability to explain socio-economic status. The combined model is a theoretical progress, which complements the traditional approaches and increases our understanding of cities and urbanisation processes.

## Introduction

The human population living in cities is predicted to increase from 50 per cent in 2008 to 75 per cent by 2030 [1]. This fast urbanisation process and consequent changes in urban systems raise concerns about the development of urban systems [2]. For example, urbanisation may affect crime rates, human health and infrastructural resilience of societies [2, 3]. Although there are numerous examples of negative impacts of urbanisation on the socio-economic status of urban systems, urbanisation can also present an opportunity to improve the livelihoods of the people inhabiting urban areas. A range of studies has shown that urbanisation can increase productivity in urban systems, resulting in, for example, higher per capita income and innovation [3, 4]. The spatial configuration of urban systems is believed to interact with urban socio-economic status in complex manners [5–7]. Several studies have demonstrated the importance of urban configuration on different aspects of human well-being [8], economy [9, 10], social sustainability, sense of place [11, 12], ecology, environment [10], transportation and accessibility [13] using qualitative [13], theoretical [14] and empirical [6, 11] methods. Despite the acknowledged importance of urban configuration, the complex nature of urban systems makes it hard to formulate the relationships between urban configuration and urban socio-economic status. However, in order to be able to steer urbanisation in a direction that profits human societies, we still need to increase our understanding of how the configuration of urban systems affects those societies’ socio-economic status [15].

In general, it is believed that urban development and configuration are driven by two processes [16, 17]. On the one hand, the bottom-up self-organisation drives the development of urban systems, which results in a universal urban scaling law [18, 19]. On the other hand, top-down regulations (e.g. regional laws and policies) and environmental conditions regulate and constrain the relationship between different areas and the consequent development of urban systems [16]. These two processes are usually studied separately and are rarely considered complementary processes to explain urban socio-economic status.

The theory of urban scaling poses that urban productivity increases with size (i.e. increasing returns to scale) [20–24], which means that larger metropolitan areas generally produce, for instance, higher per capita income, GDP and more inventions [25]. However, several studies have found that the socio-economic status of urban systems significantly deviate from those predicted by the scaling law model [26]. These deviations have been attributed to random fluctuations [27], yet other studies have found that they are linked to the local factors of the metropolitan areas [25, 26]. In the latter studies, the deviations between the predicted and the measured socio-economic status are referred to as Scale-Adjusted Metropolitan Indicator (SAMI) [25, 26]. Remarkably, these SAMIs have also been associated with scale-adjusted socio-economic variables (e.g. distribution of wealth, labour force and technology) and can therefore be used to rank urban regions [26]. Yet, the reasons for these deviations from urban scaling law are not well understood. One possible explanation for these deviations is that functional relations within and between metropolitan areas are not considered in the theory of urban scaling. It is important to consider this aspect, as the socio-economic status of a metropolitan area not only depends on its population size, but also on how main centres are embedded and connected to other centres within these areas [28–30].

In contrast to urban scaling laws, functional relations between urban centres within a metropolitan area are explicitly considered in spatial planning [31]. Particularly the concept of polycentricity considers the existence of multiple functional centres and distributed connections between centres in the organisation of urban systems. A high level of polycentricity has been associated with a more advanced socio-economic status of an urban system [32, 33]. Meijers (2007) [34] suggest that the benefits of polycentricity are caused by the synergistic relationships between urban centres of a metropolitan area. Porta et al. (2012) [29] and Lorenzen et al. (2009) [30] show that the centrality patterns and therefore polycentricity of a region facilitate the flows of innovation and cooperation (especially because of the changes in the market thresholds for creative services), which result in a more efficient governance [35]. Based on such studies, polycentricity has become a normative planning goal in many top-down spatial development plans at global, European and national scales [36, 37]. Several indicators have been proposed to measure the level of polycentricity in a region [36, 38]. Many of these indicators are calculated with, among others, the distribution of metropolitan centres in an area [32]. Some indicators explicitly consider the functional connections between an area’s metropolitan centres [28, 33, 39, 40].

In order to capture both size and connections between functional centres of an urban system, a settlement network paradigm has been suggested to study the socio-economic status of such systems [39]. Functional centres in an urban system are considered nodes and the relationships between functional centres can be translated to links. Therefore, methods from complex network science could potentially be employed to study urban systems with an emphasis on the effect of functional relationships [41]. A network measure that may be suitable as an indicator for polycentricity is the network hierarchy. Hierarchy is a global network property that is used to measure the overall dominance pattern of nodes in terms of their influence on other nodes. Different hierarchy measures have been introduced to quantify the distribution pattern of dominant nodes in a network [42]. One such measure is Global Reaching Centrality (GRC), which has been developed to reveal universal features of the hierarchical organisation of relations within a network [43]. Yet, to our knowledge, GRC has never been applied in urban system studies.

In summary, both urban scaling law as well as the level of polycentricity have been linked to the socio-economic status of urban systems. This raises the question whether these two concepts are complementary to one another or whether they are actually alternative descriptions of a single underlying process. To answer this question, we assessed whether the deviations from urban scaling law (SAMIs) are correlated to our indicator of polycentricity (i.e. GRC) and whether socio-economic variables can be better explained by a combination of both concepts. The combined model aims to explain two important variables associated with the socio-economic status of urban areas, the mean and the median income. The mean income provides an overview of the overall economic status of a metropolitan area. However, the median income is considered a more robust indicator for well-being and other development processes and is favoured over mean income in social welfare studies [44]. A comparison between the mean and median income can also uncover inequalities in metropolitan inhabitants’ income [44, 45].

In this study, we focus on metropolitan areas in Switzerland. Our settlement network consists of 2944 Swiss municipalities as nodes, which are linked by commuter flows derived from the Swiss national passenger transport model (NPVM) [46]. For each municipality, we obtained income data (i.e. mean and median income) published by the Swiss Federal Tax Administration. All the data we used are from 2010. Based on this data, we show that, complementary to urban scaling, the polycentricity indicator of settlement networks can explain personal income in metropolitan areas, which provides a possible explanation for deviations from scaling law (i.e. SAMIs).

## Results

### Delineating metropolitan areas

**Fig 1:**
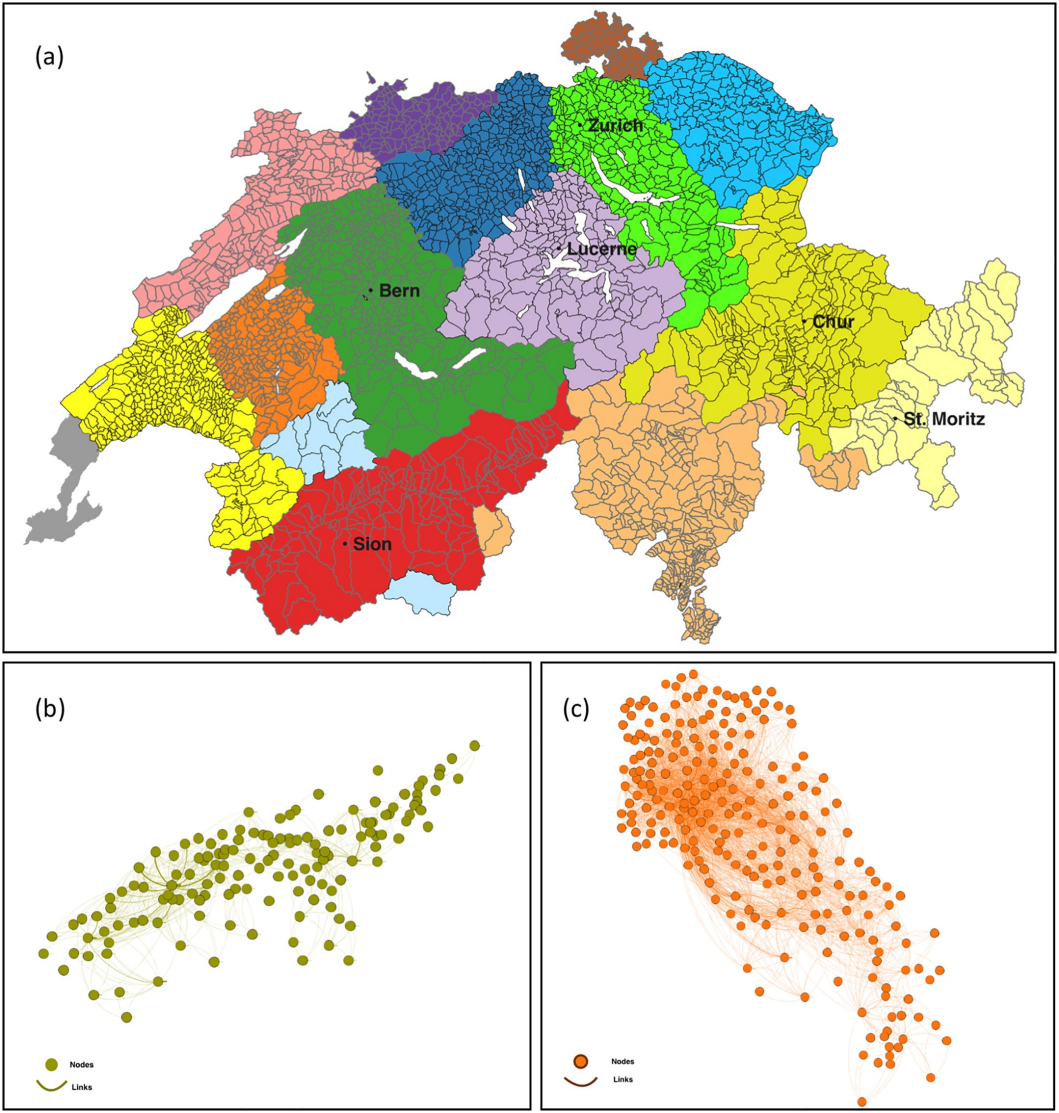
(a) Sixteen metropolitan areas in Switzerland as detected by the modularity maximisation method. Different colours are used to differentiate metropolitan areas. (b) The settlement network of the Sion metropolitan area. (c) The settlement network of the Zurich metropolitan area.

To analyse the effect of both size and functional connections on the socio-economic outcomes of metropolitan areas, we delineated metropolitan areas as the relevant socio-economic units of Switzerland. There are multiple methods to delineate urban or metropolitan areas [47–52] and the results of urban scaling studies are sensitive to the choice of method [53, 54]. We applied a community detection algorithm [55] to the settlement network and clustered municipalities based on the commuter flows between municipalities as suggested in several recent studies (for details, see Methods) [47, 56–58]. Each network community represents a metropolitan area. The commuter flows represent the functional connections between urban centres in the polycentricity concept [40]. With this approach, we identified 16 metropolitan areas in Switzerland (Fig 1a). These 16 areas reflect quite well the official labour market areas delineated by the Swiss Federal Statistical Office (FSO) and similar researches in Switzerland [59, 60] and are comparable to NUTS 3, which are European metropolitan units for spatial studies [61]. The metropolitan areas with their main statistics are shown in Table 1.

**Table 1:** Ranked Swiss metropolitan areas based on SAMI and related statistics (GRC hierarchy, Population size, Median and Mean income (CHF)).

Rank	Metropolitan area*	number of municipalities	Median income	Mean income	Population	GRC	SAMI
1	St. Moritz	32	37864	45896	20683	0.06764	0.11637
2	Zurich	228	45866	55118	3322387	0.05847	0.09762
3	Geneva	86	42609	51903	784430	0.00042	0.09488
4	Luzern	139	42535	50548	545858	0.01869	0.08284
5	Basel	145	42728	50745	1094345	0.00159	0.05909
6	Schaffhausen	51	41946	45703	93483	0	0.05221
7	Saanen	11	32857	40533	16186	0.06	0.00185
8	Aarau	327	42317	46190	512323	0.03876	-0.00479
9	Chur	167	39809	43612	148496	0.10068	-0.01302
10	Lausanne	346	39998	45994	718238	0.04250	-0.02247
11	Lugano	279	36617	45999	882693	0.13414	-0.03056
12	St. Gallen	170	40136	44536	427819	0.00592	-0.03409
13	Fribourg	254	39679	42560	219819	0.01792	-0.05302
14	Bern	369	40004	43694	1208880	0.07790	-0.09448
15	Neuchatel	198	36859	39654	217130	0.16494	-0.12326
16	Sion	142	36867	39212	190109	0.14733	-0.12919

* Metropolitan areas are named by the city with the largest population within the community.

The metropolitan areas spatially coincided with the official Swiss labour market regions [59, 60]. The metropolitan areas also correspond to the main landscape typologies of Switzerland [62, 63]. The Swiss Alps separate the southern and northern communities and have a major effect on the functional connections and the consequent community structure. In the Swiss Plateau (“Mittelland” in German), north of the Alps, there is a high population density and a large number of small municipalities which are connected through a dense transportation system, compared to the Alps and other mountainous areas in Switzerland (i.e. Jura). The Swiss Plateau covers about 30% of the surface area of Switzerland, yet accommodates almost 60% of the Swiss population. The denser settlement network in the Swiss Plateau results in smaller sized communities as detected by our algorithm. The network communities also reflect cultural similarities among communities. For instance, the cluster identified in eastern Switzerland corresponds to the area where Romansh is spoken.

Not only the community structure but also the topology of each settlement network within a community is influenced by the topography and the socio-economics of the communities. For instance, the settlement network surrounding Sion in the canton of Valais (Fig 1b) is different from the network surrounding Zurich within the Swiss Plateau (Fig 1c). The settlement networks within the Alpine region (e.g. Sion area) are developed along the valleys, because the mountain ridges in between hamper the development of direct links between nodes. Many smaller sized nodes (where the main activities are agriculture and tourism) are connected to the nearest larger node, which results in a hierarchical connection pattern. This is not the case in the area surrounding Zurich, where there are several connected hub nodes (i.e. multiple economic centres) resulting in a less hierarchical connection pattern. The network topology within the metropolitan areas thus affects the measured hierarchy. The measured GRC ranged from 0, where there is a fully connected network (i.e. Schaffhausen), to 0.16 in an area with a strong hierarchical structure (i.e. Neuchatel; Table 1).

### Conventional scaling law and population-income relationship

According to the conventional urban scaling law, the socio-economic status of metropolitan areas is related to their population size with the equation:

$$Y(\sigma )=Y_{0}\;{N(\sigma )}^{\beta -1}$$ (1)

where Y(σ) is a per capita metropolitan indicator (e.g. per capita income, patents, energy consumption) of metropolitan region σ, Y_0_ is the normalisation constant, N(σ) is a region’s population size, and β is the scaling coefficient. Applying a logarithmic transform, Eq (1) takes a linear form (for details, see Materials and methods). Based on the value of β, three different types of scaling relationships between population size and socio-economic indicators can be observed: a coefficient of 1 results in a linear relation, a coefficient higher than 1 shows a super-linear scaling and a coefficient less than 1 shows a sub-linear scaling.

**Fig 2:**
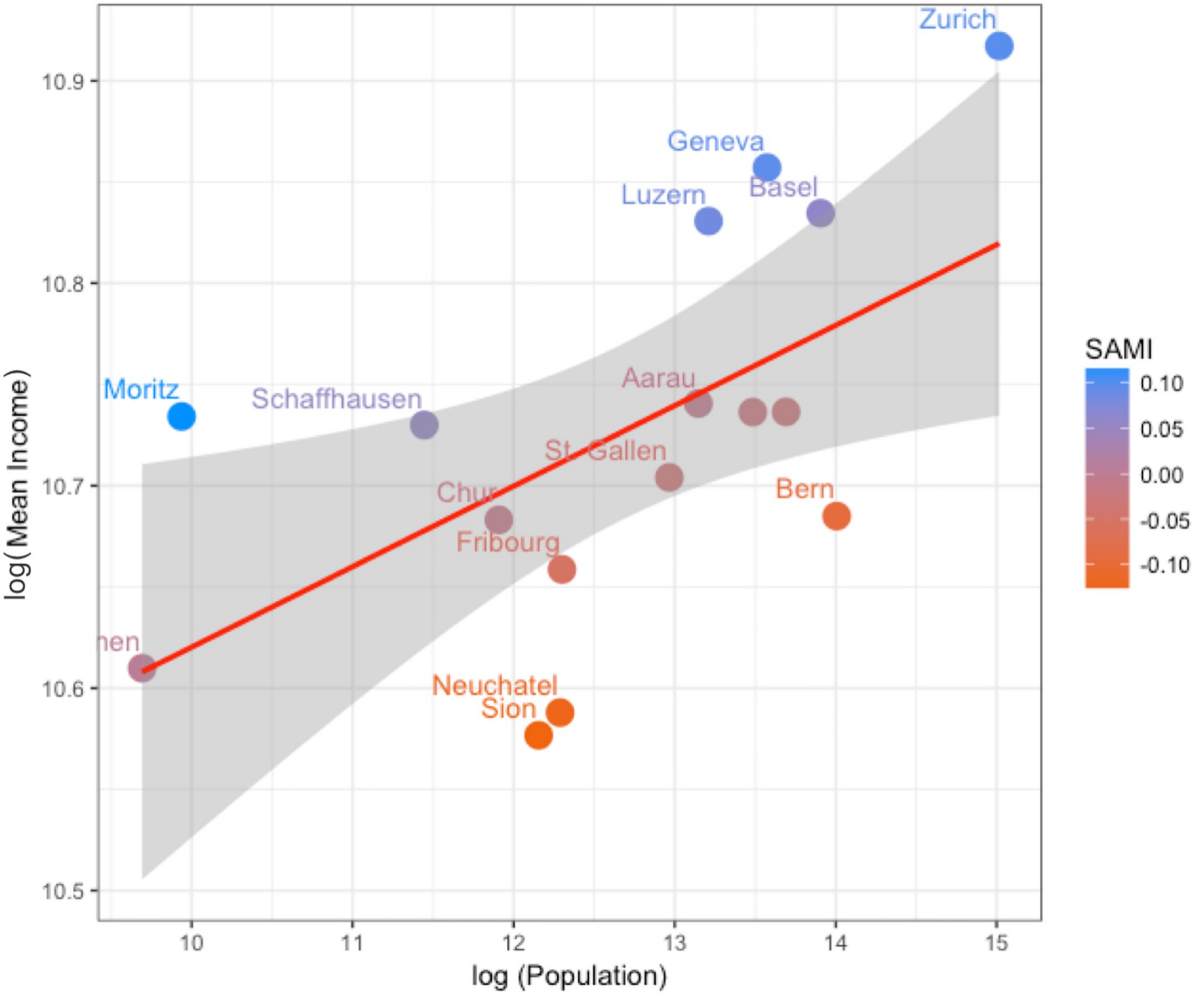
The relation between mean income and the population size of the metropolitan areas in Switzerland where β = 1.04 and Y_0_ = 27447. The grey area indicates the 95% confidence interval. SAMI is the deviation from scaling law, which is used for ranking urban areas.

In order to relate population size in the metropolitan areas to mean per capita income, we performed a regression analysis between log(Income) and log(Population) revealing a significant correlation (p < 0.05, Adj-R2 = 0.30; Table 2) (Fig 2). We estimated that the income per capita in Switzerland follows a super-linear scaling form with β = 1.04±0.01 (Fig 2). According to this correlation, the mean income would increase by 2.8% with a doubling of the population size in metropolitan areas. This increase in per-capita income is known as an inherent characteristic of urban systems, which has been hypothesized to drive the urbanisation process and is a motivation for individuals to move to larger metropolitan areas [64]. The β-coefficient detected in this study is smaller than that detected in a global analysis in which β was found to range between 1.09 and 1.13 with a confidence level of 95% [3] and, is more similar to that estimated in Western-European cities (β = 1.03±0.05) [16]. Yet, whereas Strano (2016) [16] found that their estimated β-coefficient was not significant and concluded that there was no super-linear scaling in Western-European cities, we found our β to be significant and conclude that there is super-linear scaling in Switzerland. Following Leitao et al. (2016) [27], we also compared the Bayesian Information Criterion (BIC) value of the estimated model to a null-model where the coefficient is set to zero (β-1 = 0; linear scaling). We found a ΔBIC value of 4.06, which indicates that there is a positive support for the super-linear scaling hypothesis (Table 2).

**Table 2:** Statistical analysis of different models using main explanatory variables.

Model comparison						
models	estimated(log(*y*_0_),(β-1),α)	Adj-R2	p-value(s)*	standard errors	BIC	Δ*BIC*_null_**
1	**Mean income = f(*Population***^***β*−1**^**)**					
	10.22, 0.0397	0.301	0.016	0.1854, 0.0146	-28.89	4.06
2	**Mean income = f(*e***^α*Hierarchy^**)**					
	10.79, -1.0178	0.275	0.022	0.0309, 0.3933	-28.31	3.48
3	**Mean income = f(*Population***^***β*−1**^ * *e*^α*Hierarchy^**))**					
	10.33, 0.0356, -0.9036	0.537	0.011, 0.014	0.1553, 0.0119, 0.3165	-33.90	9.08
4	**Median income = f(*Population***^***β*−1**^ * *e*^α*Hierarchy^**))**					
	10.19, 0.0351, -0.7368	0.653	0.001, 0.007	0.1128, 0.0087, 0.2299	-44.14	13.69

* p-values are reported for individual explanatory variables; both explanatory variables are significant in the combined models.

** BIC difference with the null model in which the model parameters *β* − 1 and *α* are set to 0

A second product of the scaling analysis is the construction of scale-adjusted indicators of metropolitan performance. According to recent studies [25, 26], both the scaling exponent as well as the deviations from the scaling law are important for understanding the socio-economics of metropolitan areas. Derived from Eq (1), SAMIs can be calculated with the following equation (for details, see Materials and methods):

$$SAMI\;(\sigma )=\text{ln}\left(\frac{Y_{\sigma }}{Y_{0}N_{\sigma }^{\beta -1}}\right)$$ (2)

where Y_σ_ is the value of a per-capita metropolitan indicator (e.g. mean income) of metropolitan region σ, and Y_0_, N(σ) and β are the same as in Eq (1). SAMIs should be zero in a hypothetical metropolitan system where the scaling law is an ideal predicting model. In our study region, clear deviations were identified between reported income and that predicted by the urban scaling law (Fig 2). We ranked Swiss metropolitan areas by their SAMI (Table 1). In Switzerland, SAMIs range between -0.129 for the Sion metropolitan area to 0.116 for the St. Moritz metropolitan area (Table 1). Metropolitan areas with relatively high (e.g. St. Moritz, Zurich, Geneva) or low (e.g. Sion, Neuchatel, Bern) SAMIs are the outliers in Fig 2.

### SAMIs explained by network hierarchy

**Fig 3:**
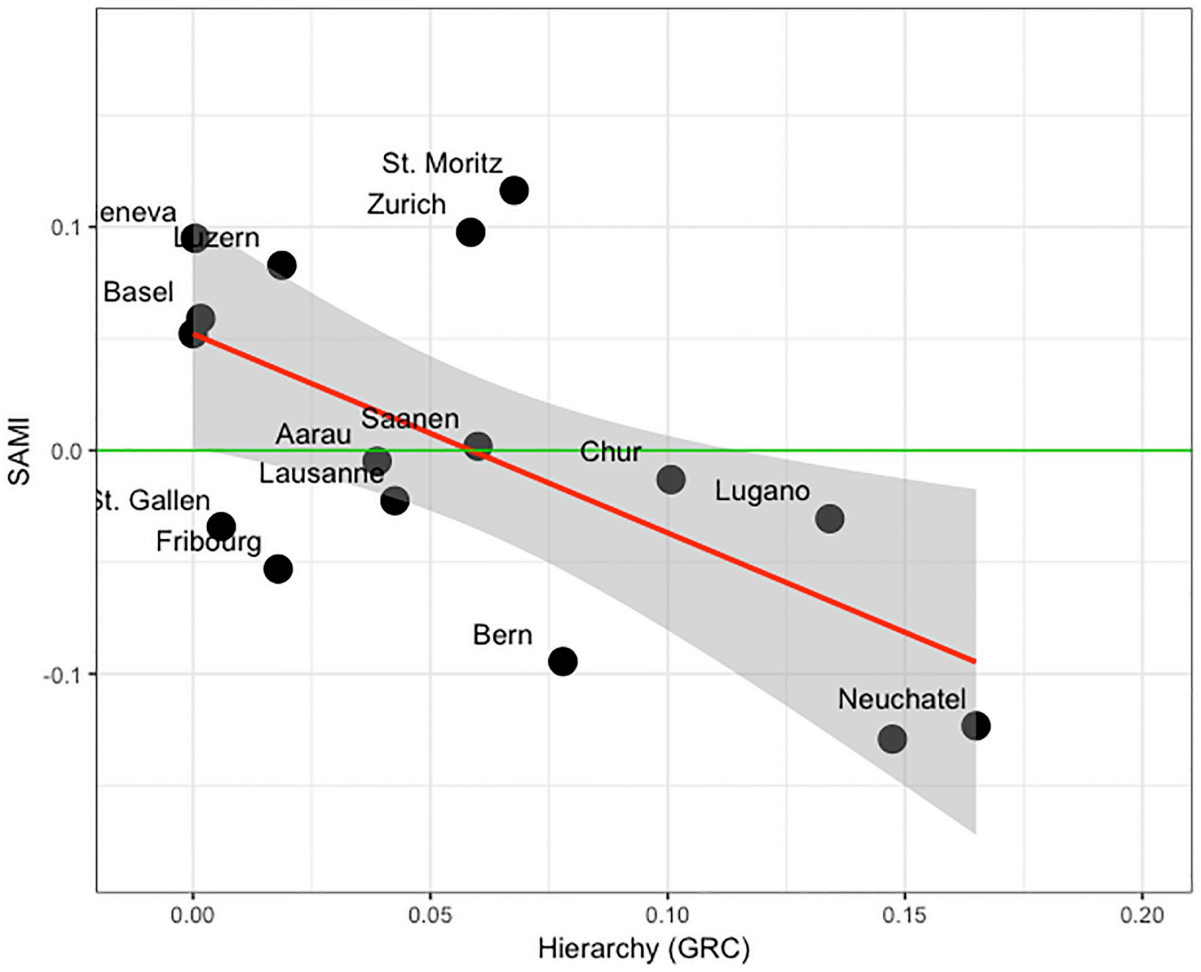
The relationship between hierarchy of settlement network and SAMIs. The red line is the ordinary least squares fit to data points and the green line shows the reference line (SAMI = 0). Metropolitan areas above the green line are associated with a higher economic outcome than expected from urban scaling, while metropolitan areas below the green line have a lower than expected outcome. The grey area indicates the 95% confidence interval.

We subsequently analysed the associations between the hierarchical structure of metropolitan settlement networks and their SAMIs. Hierarchy (GRC) is used to measure the heterogeneity of the local reaching centralities (*C*_*R*_) of the municipalities within a metropolitan area. *C*_*R*_(*i*) is equal to 1 if the *i*th municipality can be reached directly from all other municipalities within a metropolitan area (i.e. municipality *i* is very central). If a municipality in the settlement network can be reached only via other municipalities, then it would be less central and *C*_*R*_(*i*) < 1 (as can be seen in the simple network model in the “Materials and methods” section). We calculated GRC for each metropolitan settlement network (Table 1). We found a significant correlation (p < 0.05, Adj-R2 = 0.34; Table 2) between GRC and SAMIs (Fig 3). Some alpine metropolitan areas like Sion and Chur have relatively high GRC and negative SAMIs (Fig 3), while many of the metropolitan areas (e.g. Geneva, Zurich, Lucerne) have relatively low GRC and positive SAMIs. A special case in our results is the Bern metropolitan area, which is more hierarchical and has a lower than expected income (negative SAMI) than other metropolitan areas in the Swiss Plateau with a similar population size, such as Basel. These results provide a bridge between the two concepts of urban scaling law and polycentricity, which we further assess in the next section.

### Multivariate analysis

**Fig 4:**
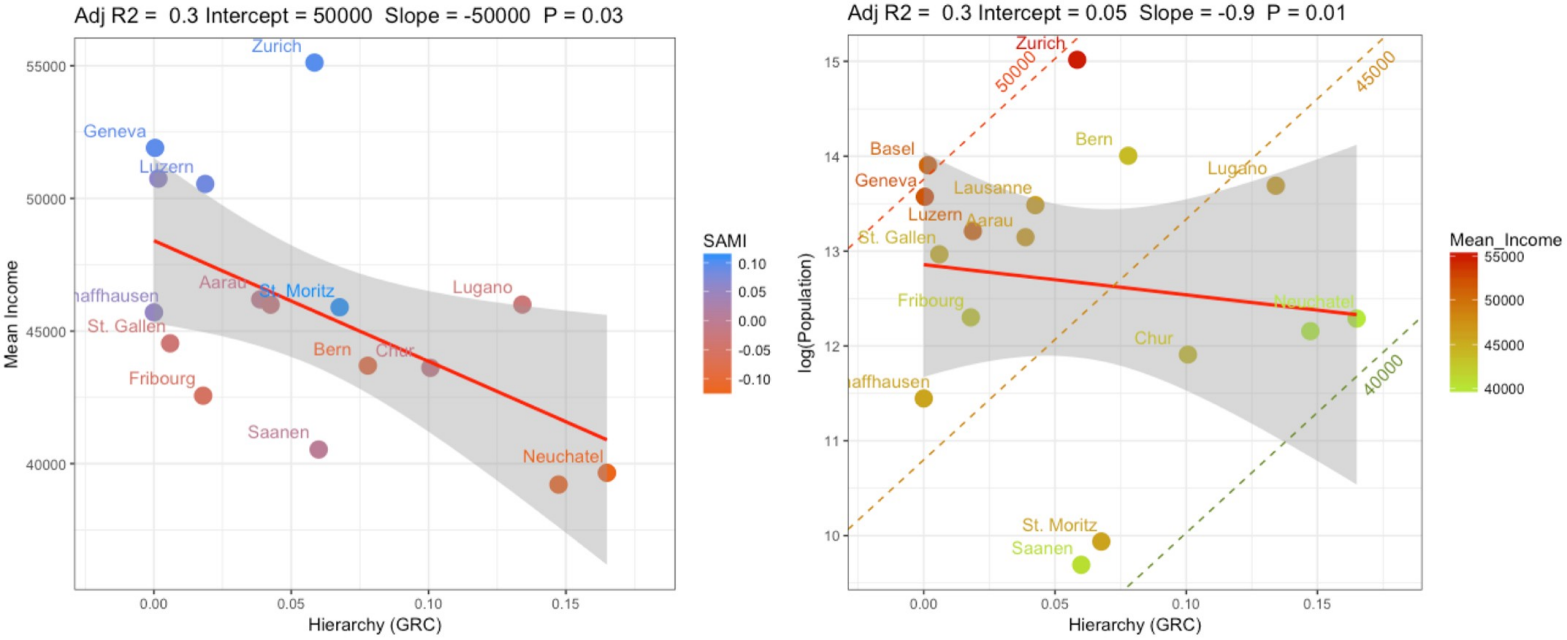
(a) Scatter plot showing the relationship between mean personal income and metropolitan hierarchy (GRC). SAMIs are shown with a colour gradient. (b) Scatter plot showing the interdependence of two explanatory variables. The isoclines represent different mean income levels (i.e. 40’000, 45’000 and 50’000 CHF). The colour gradient shows the mean income of metropolitan areas.

We used a regression analysis to assess the extent to which two explanatory variables (i.e. population size and GRC) explain mean income in Swiss metropolitan areas. The multivariate analysis consists of three steps: First, we assessed the relationship between GRC and personal mean income (Fig 4a). Second, we analysed the interdependence between the two explanatory variables (Fig 4b). Third, we formulated a multivariate regression model to assess the combined effect of both metropolitan size and GRC on mean incomes. We used the BIC to compare the combined model with individual bivariate regression models (i.e. Income~GRC and Income~Population) as well as with null-models in which all the coefficients are set to zero (β-1 = 0, α = 0) [27].

We found a significant negative correlation between GRC and the logarithm of mean income (p < 0.05, Adj-R^2^ = 0.28; Fig 4a). Due to the existence of 0-values, we did not log-transform the GRC values. A comparison of the BIC scores between models with and without log-transformed GRC values showed that differences were small (-33.9 and -32.96, respectively). Fig 4a shows that a 0.1-unit decrease in the hierarchy of the metropolitan network (i.e. increased polycentricity) is associated with approx. 5000 CHF increase in personal mean income per year across Swiss metropolitan areas. From Fig 4b we observe that metropolitan hierarchy is independent of metropolitan size (p > 0.05, Adj-R^2^ = -0.06). Given that both metropolitan size as well as metropolitan hierarchy were significantly correlated to mean income (Figs 2 and 4a) but mutually uncorrelated (Fig 4b), allowed us to combine them both in a multivariate regression model. The following models were fitted:

$${Mean\;income}_{\sigma }=30638*{Population\;size(\sigma )}^{0.0356}*e^{-0.9036({GRC}_{\sigma })}$$ (3)

$${Median\;income}_{\sigma }=26635*{Population\;size(\sigma )}^{0.0351}*e^{-0.7368({GRC}_{\sigma })}$$ (4)

**Fig 5:**
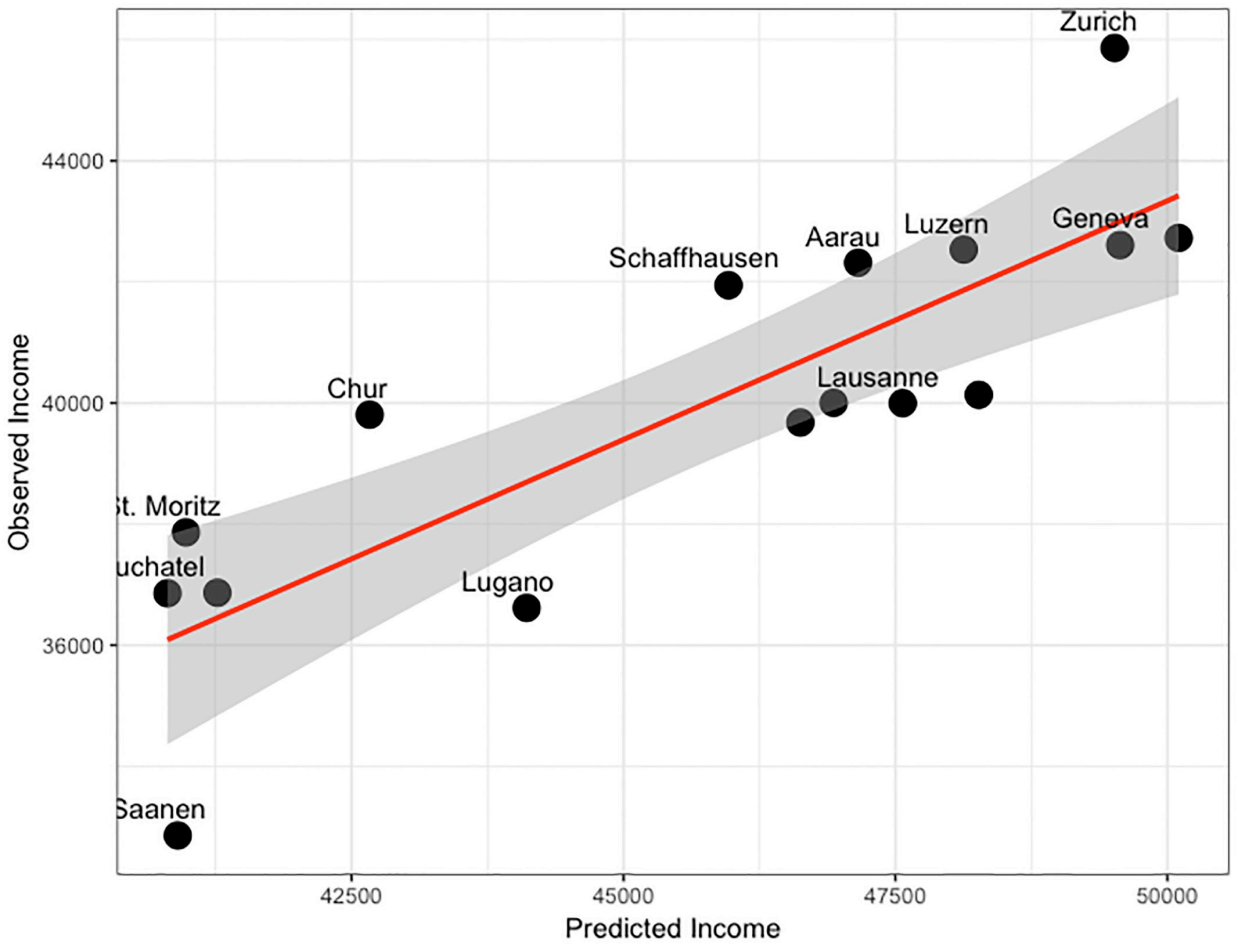
Comparison of modelled median income and observed median income.

The mean income model (Eq 3) had a good explanatory power (Adj-R^2^ = 0.54) and both explanatory variables were significant (p < 0.01; Table 2). The low BIC of the combined model (-33.9) compared to that of the univariate models (BIC > -28.9; Table 2) indicates that the combined model better predicts and explains personal income in metropolitan areas. The two univariate models had a positive evidence against their null-models (Δ*BIC*_*null*_>2; Tables 2 and 3), whereas the multivariate model had a strong evidence against its null-model (Δ*BIC*_*null*_>6; Tables 2 and 3). The median income model (Eq 4) had a higher model fit than the mean income model (Adj-R2 = 0.65) and a very strong evidence against its null-model (Δ*BIC*_*null*_ >10; Tables 2 and 3). For this latter model, we also show that the predicted median income corresponds well to the observed median income (Fig 5).

**Table 3:** The scale of ΔBIC in the model comparison as suggested by Kass and Raftery [96].

ΔBIC	Evidence against higher BIC
0 to 2	Not worth more than a bare mention
2 to 6	Positive
6 to 10	Strong
>10	Very strong

The combined relationship between size and polycentricity reveals interesting trade-offs between metropolitan size and polycentricity. The isoclines in Fig 4b represent different mean income levels (i.e. 40’000, 45’000 and 50’000 CHF). The existence of such economic isoclines suggests that smaller sized metropolitan areas can achieve a higher economic status compared to the same-sized metropolitan areas by increasing their level of polycentricity.

In order to test the robustness of the combined model against the modularity maximisation algorithm, we assessed the model fit (adjusted R-squared of the combined model) for different modularity scores (i.e. the quality of the community detection is linked to the modularity score). Our analysis shows that there is a positive and highly significant correlation between adjusted R-squared of the combined model and the modularity score of the delineated metropolitan areas (S1 Fig). This suggests that a better quality of the modularity maximisation algorithm results in a better model fit of the combined model.

## Discussion

In this study, we found strong effects of settlement patterns on the socio-economic status of metropolitan areas. These effects have important consequences for the planning and monitoring of settlement developments. Two major theories for studying settlement patterns are the urban scaling law [3, 25] and polycentricity [28, 34, 36]. These two theories were developed based on different assumptions and applied for different purposes. For example, polycentricity was the basis for formulating a global urban development goal [38] and urban scaling has been used to understand historic development processes [16]. Although the urban scaling law is based on strong theories, it does not consider functional relations between centres (connection pattern of centres) within a metropolitan area, which makes the theory difficult to apply to urban planning [39, 65]. The polycentricity concept, however, has been especially developed for planning purposes [38] and mostly to enhance socio-economic development of metropolitan areas [66]. Yet, in practice it is still considered to be a vague planning goal [67], mostly due to the lack of a solid theoretical foundation [36]. In this article, we have shown that these two concepts are complementary and can be combined in a well-founded model for planning purposes. By using the extended model, monitoring polycentricity in metropolitan areas becomes possible.

We found that both the population size as well as the hierarchical connection pattern of centres in metropolitan areas well explained the variation in mean income in metropolitan areas in Switzerland (Figs 2 and 4a). Variations in median income were even better explained by these two variables. Although the β of the combined median income model cannot be used to make statements about urban scaling relationships, this model is capable of predicting an important indicator of a region’s social-economic status. Median income is often preferred over mean income as an indicator for social-economic status [44]. As the income distribution is positively skewed in most regions, the mean income is usually influenced by changes in the long tail of the distribution (i.e. the few very high-income individuals). In contrast, the median is less sensitive to such changes and better reflects the income of the majority of the population. Therefore, the difference between the mean and median income can shed light on the income inequality of a region [44]. Comparing predictions from the mean and median income models, could thus also be used to assess a region’s income inequality [45]. However, this analysis is beyond the scope of this manuscript.

Hierarchy is a fundamental characteristic of complex systems (e.g. social, biological and metabolic systems) [68–71] and it is associated with efficiency and stability of these systems [72, 73]. Although different indicators of hierarchy have been developed so far [42, 74], improving hierarchy indicators is still an important challenge in many research fields such as urban planning. Particularly the investigation of the emergence of hierarchy in systems is still a research field in its infancy. One possible explanation for this phenomenon in urban systems is that hierarchy emerges because of resource constraints and local interactions aiming at decreasing the costs of the system and increasing its stability [75–78]. Although we have not studied the mechanisms behind the emergence of hierarchy in metropolitan areas, our findings may trigger research to uncover such mechanisms. An import research topic of the recent years is the prediction of critical transitions in systems [79, 80]. Due to the fact that hierarchy is responsive to changes in local interactions [81, 82], it can potentially also be used for anticipating critical transitions in metropolitan areas.

In our study, the hierarchy was correlated to SAMIs (Fig 3), which have been found to reflect local factors affecting socio-economic outcomes of different metropolitan areas [26]. The fact that SAMIs can be related to important properties of metropolitan areas also sheds light on the deficiencies of urban scaling. The urban scaling theory assumes that the population size is the main factor influencing economic outcomes of a metropolitan area [3], which is in strong contrast to what we can learn from, for instance, Swiss alpine regions. Swiss alpine metropolitan areas are socio-economic entities with a special type of social and economic structure [62]. In these regions, tourism and agriculture are two important economic activities, which cause seasonal changes in the population of alpine metropolitan areas are not reflected in an area’s population size, but have important socio-economic consequences. Another example is the Bern metropolitan area, which is very different in terms of major economic activities in comparison with other metropolitan areas on the Swiss Plateau [83], as it hosts most of the government agencies. Although such local factors will usually not affect the population size of these metropolitan areas, they can change the functional connection pattern of the centres within these areas [84]. By making use of the hierarchy, we were able to incorporate this pattern of functional relation between centres in our extended model, leading to a better model fit and a strong reduction in the model residuals.

The network of urban flows, such as commuter flows, proved to be useful in revealing the dynamic spatial structures of metropolitan areas, which was also found by Zhong et al. [58]. Shen and Batty [57] have shown that modularity maximisation in networks of commuter flows is an effective approach to delineate perceived metropolitan areas. The delineated boundaries reflect the relatedness of places in terms of their functionalities. Earlier trials to define metropolitan areas based on aggregated data (like settlement density) [53] did not consider such local interactions. In contrast to administrative boundaries, which remain mostly constant over time, commuter flows are related to changing socio-economic conditions [85–89] and can thus be used to delineate metropolitan areas in a more dynamic way. Community detection with modularity maximisation aims to maximise the connectivity within a community and minimise the connections between the communities. This way the spatial correlations between the metropolitan areas are also minimised. This is important, because most regression or correlation analysis assumes independent observations. If we would have used, for instance, administrative boundaries or size criteria for the delineation of cities in Switzerland, two neighbouring cities would be considered as independent observations, while in reality they could be highly connected and functioning as a single entity. This would bias the fitted models (The effect of quality of the community detection on the combined model is shown in the supplementary material).

Although a settlement network of commuter flows was used in our study, different types of metropolitan flows (e.g. number of calls, number of emails, financial transactions) could also be considered to measure the hierarchical organisation of metropolitan areas. A drawback to using flows is that there can be strong boundary effects caused by flows outside the study area. For instance, we chose to perform our study in Switzerland, but there are many trans-border commutes. Geneva, for example, is highly connected to France. Although disregarding these trans-border commutes potentially biased our results, we did not consider international commutes, mainly due to a lack of comparable data. Although other studies found that the detectability of scaling relationships was sensitive to the definition of boundaries [53], we have shown that our extended model is fairly robust to changes in the delineation of metropolitan areas. We found comparable model fits for different settings in the modularity maximisation algorithm leading to different borders of metropolitan areas in Switzerland. Despite this robustness, the fairly small sample of 16 metropolitan areas in this study can be a source of bias. Further testing should be performed to determine the robustness of our results to larger numbers of metropolitan areas. We here used an algorithm that has been successfully used in other studies to delineate metropolitan areas (Shen & Batty) [57]. Yet, it would be worthwhile to perform a comparative analysis of such algorithms to determine the most suitable and robust algorithm for this purpose [59].

The two input variables (population size and hierarchical organisation) in our extended model can be compared to economic resources for productivity of a region [25]. From the results of our study, we hypothesize that the organisation of centres can be considered a valuable economic resource for metropolitan areas. Another advantage of the combined model is that it provides a quantitative tool to understand trade-offs between economic resources, which allows us to measure the sensitivities of economic outcomes to changes in transportation or population growth in an urban system. In other words, our findings suggest that both “economy of scale” and “economy of organisation” should be considered for studying the economy of urban areas. The final model provides a good link to production functions in economic theory and there is still much potential for developing this research further. In particular, an interesting line of research would be to study the role of technology, innovation and demographic developments in the emergence of hierarchical patterns in metropolitan areas [90]. Still, the transferability of our approach to other regions should first be assessed to determine the generality of our findings.

For future generations, the socio-economic consequences of the ongoing global urbanisation largely depend on current urban planning decisions. Choosing between centralisation and decentralisation is a big dilemma in planning. The bottom-up process of urban development may result in a more monocentric and more hierarchical urban structure, because people tend to be drawn to larger cities for higher individual socio-economic gains [91]. That is one of the reasons why we have witnessed a fast urbanisation and rural abandonment in the last decades [92]. However, as was apparent from our study, a polycentric urban configuration may result in better socio-economic gains for its inhabitants. Some organisations, such as the European Spatial Planning Observation Network and the United Nations, suggest using top-down planning instruments to steer metropolitan areas towards more polycentric settlement patterns [9, 38]. In Europe and in other continents, debates are taking place about the amounts that should be invested in decentralisation and in fostering relationships between different inter- and/or intra-border metropolitan areas [93]. Previous theories have been vague or not quantitative enough for actual estimations of investment amounts for decentralisation of metropolitan areas. With the combined model presented here, one can make quantitative predictions about the economic gain that can be expected with certain changes to the settlement network and its population size.

## Materials and methods

### Case study area

Our case study area is Switzerland, which consists of three major regions characterised by different types of landscapes. The densely populated Swiss Plateau is fringed in the north by the Jura Mountains and in the south by the Alps (see Kienast et al. (2015) for details [94]). This typology is usually used to differentiate the main characteristics of the Swiss landscape [94]. The Swiss Plateau is densely populated and has a well-developed transportation system, whereas the Jura and Alpine areas are sparsely populated.

The Swiss landscape is not only differentiated by landscape typology but also by language regions with their specific cultural and ecological heritage. Four different languages are spoken in Switzerland. In the eastern part of Switzerland mainly German is spoken, while in the western part the main language is French. Italian and Romansh are spoken in the Canton Ticino, south of the Alps, and in the canton of Grisons in the south-east, respectively. The socio-economic status of these regions may be affected by regional specific factors, which can also affect the functional relationships within and between these areas.

In our study, we constructed a network for the case-study area. The nodes in this network were the zones in the Swiss National Model for Passenger Transport (NPVM), corresponding to the municipalities (except in the large cities which are divided in multiple zones [46]). The weights of the links are the number of daily commutes between municipalities on workdays. The weighted network was used for the community detection and the non-weighted network was used for the calculation of the GRC hierarchy measure. The data underlying this network was obtained from the 2010 population census of Switzerland from the Swiss Federal Statistical Office and NPVM from the Swiss Federal Office for Spatial Development.

We used income data for municipalities published by the Swiss Federal Tax Administration. In the few cases where municipalities were divided into several NPVM zones (i.e. in the larger cities), we assigned the same income data from the municipality to each of the NPVM sub-zones.

### Community detection

We used the modularity maximisation method (Louvain algorithm) [55] to detect communities in our network based on the commuter flows as suggested in [47, 56–58]. For this purpose, we used the python-louvain package on pypi in Python 3.0. This algorithm maximises the modularity Q:

$$Q=\frac{1}{2m}\sum_{i,j}\left[A_{ij}-\frac{k_{i}k_{j}}{2m}\right]\delta (\sigma_{i},\sigma_{j})$$ (5)

where *A*_*ij*_ is the weight of the link between nodes *i* and *j* (number of commuters between nodes *i* and *j*), *k*_*i*_ is the sum of all the links to node *i*, *σ*_*i*_ is the community in which node *i* is located, the function *δ*(*σ*_*i*_, *σ*_*j*_) is equal to 1 if both nodes *i* and *j* are located in the same community and otherwise it is equal to 0, and finally $m=\frac{1}{2}\sum_{i,j}\left[A_{ij}\right]$.

The algorithm uses a greedy optimisation procedure to increase the modularity value by changing the partition structure in an iterative process. Each detected community was considered a metropolitan area. The income data for the detected metropolitan areas were calculated by aggregating the income data of individual zones.

### Global Reaching Centrality (GRC)

To quantify the hierarchy within a metropolitan area, we used the Global Reaching Centrality (GRC), developed by E. Mones [43]. This measure is a parameter-free indicator for which no a priori metrics need to be defined. GRC can be used for all types of directed or undirected and weighted or unweighted networks, which makes it useful in numerous applications [43].

In order to calculate GRC, we first calculated the local reaching centralities of municipalities within a metropolitan area. Local reaching centrality, *C*_*R*_(*i*), is a normalised centrality measure of node *i* within a metropolitan area (which can be a value between 0 and 1). *C*_*R*_(*i*) is calculated as follows:

$$C_{R}(i)=\frac{1}{N-1}\sum_{j:0<d(i,j)<\infty }^{}\frac{1}{d(i,j)}$$ (6)

where *d*(*i*, *j*) is the number of steps through the network to reach node *j* from node *i*. If all the nodes in a network are reachable from node *i*, then *C*_*R*_(*i*) is 1, which is the maximum possible value for local reaching centrality and if the node *i* is not directly reachable from other nodes, *C*_*R*_(*i*) decreases.

The heterogeneity of local reaching centralities in a network is measured by GRC, which is calculated as follows:

$${GRC}_{\sigma }=\frac{\sum_{i\in V_{\sigma }\;}\;[C_{R}^{Max}-C_{R}(i)]}{N-1}$$ (7)

where $C_{R}^{Max}$ is the maximum local reaching centrality amongst all the nodes in the metropolitan area σ, N is the number of nodes in the network, and *V*_*σ*_ is the set of all nodes within σ [43].

**Fig 6:**
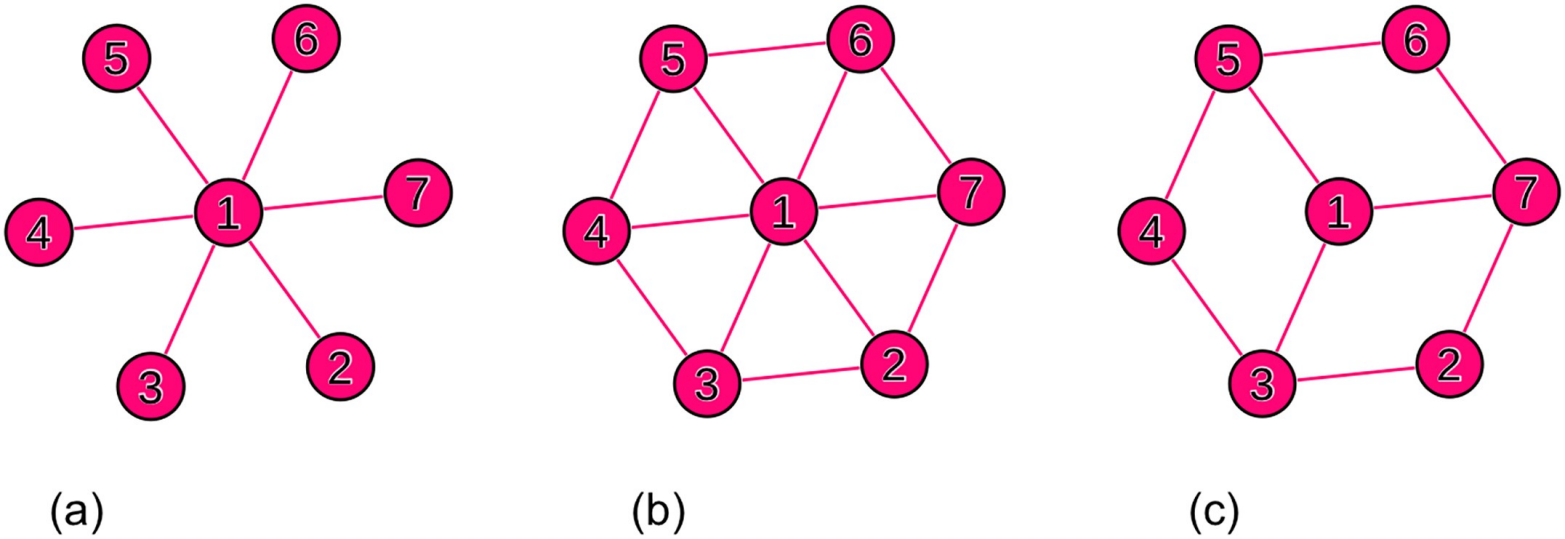
An illustration model to show different connection patterns in settlement network and relative changes in Global Reaching Centrality (GRC): (a) GRC = 0.42, (b) GRC = 0.25, (c) GRC = 0.07.

Making use of simple hypothetical networks, we showed that a settlement network with one central node (i.e. monocentric) had a high hierarchy level, while a network with multiple central nodes (i.e. polycentric) had a lower hierarchy measure (Fig 6).

In a monocentric network (Fig 6a), where all peripheral nodes are connected to a central node but not to each other, the network hierarchy was relatively high (GRC = 0.42). In a second case (Fig 6b), where peripheral nodes are connected to each other directly, the GRC decreased to 0.25. This is in agreement with a higher perceived polycentricity level, although a central node was still recognisable in this network. In the last case (Fig 6c), connections were more equally distributed between the nodes, which resulted in a polycentric network where no central or peripheral node is recognisable (i.e. any of the nodes 1,3,5 and 7 could be a central node). The GRC in this network was 0.07, which is the lowest GRC amongst the three networks. This analysis of hypothetical networks supports our thesis that levels of polycentricity can be measured with GRC.

### Calculation of the incomes for metropolitan areas

We used the following equation to calculate the mean income of the metropolitan areas:

$${Mean\;income}_{\sigma }=\frac{\sum_{i\in V_{\sigma }}{Mean\;income}_{i}*{Population}_{i}}{{Population}_{\sigma }}$$ (8)

where, *Mean income*_*i*_ is the mean income in municipality *i*, *Population*_*i*_ is the population in municipality *i*, and *Population*_*σ*_ is the total population in metropolitan area σ.

The median income in each metropolitan area could not simply be derived by aggregating the mean or median incomes from municipalities. Therefore, had to first reconstruct the personal incomes in each municipality. We assumed that the income distribution follows a log-normal distribution function as this is the case in many regions [45, 95]. We reconstructed the inhabitants’ income distribution for each of the 2944 municipalities. The probability density function (pdf) of a log-normal distribution is calculated by:

$$f(x)=\frac{1}{\sqrt{2\pi }{\;\sigma }_{i}x}\text{exp}\left(-\frac{{\left(ln\left(x\right)-\mu_{i}\right)}^{2}}{2{\sigma_{i}}^{2}}\right)$$ (9)

Since we have the median and mean of the income data for each municipality we were able to calculate the pdf parameters (*μ*_*i*_ and *σ*_*i*_) for each municipality as follows:

$$\mu_{i}=\text{log}\left({Median\;income}_{i}\right)$$ (10)

$$\sigma_{i}=\sqrt{2*\text{log}(\frac{{Mean\;income}_{i}}{{Median\;income}_{i}})}$$ (11)

where *Median income*_*i*_ is the median income of municipality *i*.

With μ_*i*_ and *σ*_*i*_ the pdf of each municipality was reconstructed. We drew *p*_*i*_ samples from each pdf, where *p*_*i*_ is the working population size of municipality *i*. To calculate the *median income*_*σ*_ of metropolitan area *σ* all the samples from the municipalities in a metropolitan area were aggregated:

$${Median\;income}_{\sigma }=median\;\{{⋃}_{i\in V_{\sigma }}{Median\;income\;set\;}_{i}\}$$ (12)

where the *Median income set*_*i*_ is the set of sampled personal incomes in the municipality *i*.

### Urban scaling

In this study, we compared how well the mean income per metropolitan area could be explained by the area’s population size and the GRC. The relationship between population size and income is given with the urban scaling law:

$$Y^{\prime }(\sigma )=Y_{0}^{\prime }\;{N(\sigma )}^{\beta }$$ (13)

where *Y*′(*σ*) is the total personal income of metropolitan area *σ* and $Y_{0}^{\prime }$ is the normalisation constant. To obtain the per-capita urban characteristic (e.g. per-capita income), we divided Eq (13) by the population size of urban area *σ* (*N*(*σ*)), which results in the following equation:

$$Y(\sigma )=Y_{0}\;{N(\sigma )}^{\beta -1}$$ (14)

where *Y*(*σ*) is the per-capita mean income, *Y*_0_ is the new normalisation constant and *N*(*σ*) is the population size of urban area *σ*. The new scaling coefficient for the per-capita characteristic is *β* − 1, where *β* is the same coefficient as in Eq (13).

Using a logarithmic transformation, the equation takes a linear form as:

$$\text{log}(Y(\sigma ))={\text{log}(Y}_{0})+(\beta -1)*\text{log}(N(\sigma ))$$ (15)

Deviations of real per-capita values of a certain characteristic and those predicted by the scaling law (Eq (14)) are referred to as SAMIs. Including these deviations in Eq (15) results in the following model:

$$\text{log}(Y(\sigma ))={\text{log}(Y}_{0})+(\beta -1)*\text{log}(N(\sigma ))+\mu_{\sigma }$$ (16)

where *μ*_*σ*_ is the SAMI for metropolitan area *σ*. SAMIs can be calculated by re-writing Eq (17):

$$SAMI\;(\sigma )=\text{log}\left(\frac{Y_{\sigma }}{Y_{0}N_{\sigma }^{\beta -1}}\right)$$ (17)

We calculated the SAMIs for all the metropolitan regions and related them to the mean personal income with linear regression.

### Combined model

In our final combined model, log(*Y*(*σ*)) was a function of two variables: the logarithm of the population and the hierarchical organisation of the network. This combined model can be formulated as:

$$\text{log}(Y(\sigma ))={\text{log}(Y}_{0})+(\beta -1)*\text{log}(N(\sigma ))+(\alpha *GRC)+\mu_{\sigma }^{\prime }$$ (18)

where $\mu_{\sigma }^{\prime }$ are the residuals, and *α* is the coefficient of GRC in the model.

Using an exponential transformation, we reformulated the combined model in the form of a new scaling relationship:

$$Y(\sigma )=Y_{0}\;{N(\sigma )}^{\beta -1}e^{\alpha ({GRC}_{\sigma })}e^{\mu_{\sigma }^{'}}$$ (19)

With a perfectly fitting model $\mu_{\sigma }^{\prime }=0$, the model can be written as:

$$Y(\sigma )=Y_{0}\;{N(\sigma )}^{\beta -1}e^{\alpha ({GRC}_{\sigma })}$$ (20)

which shows the relation between per capita income and two major variables.

### Bayesian information criterion (BIC) for model comparison

Bayesian information criterion (BIC) is an indicator to compare the quality of different models with one another [96–98]. BIC is calculated using the following equation:

$${BIC}_{m}=-2\;\text{ln}\;L_{m}+k_{m}\;\text{ln}\;N$$ (21)

where $L_{m}$ is the likelihood of the model *m*, *k*_*m*_ is its degree of freedom and *N* is the sample size (number of observations). BIC is suggested as a model comparison criterion in studying scaling relationships [27].

In order to compare two models, we calculated the Bayes factor as suggested in [96] and as used in [27]:

$$B_{12}=P(data|m=2)/P(data|m=1)$$ (22)

where *P*(*data*|*m*) is the probability of observing the data given the model. *B*_12_ can be estimated using the following equation:

$$B_{12}\approx \;e^{\Delta BIC/2}$$ (23)

Based on the Eq (23), it can be shown that, for instance, a Δ*BIC* = *BIC*_1_ − *BIC*_2_ = 6 implies that the model m = 2 is almost 20 times more likely to be the right model than m = 1.

Kass and Raftery [96] suggest that with ΔBIC > 2 there is a positive support for the model with the lower BIC (Table 3).

## Supporting information

S1 Fig The relationship between combined model adjusted R- squared and modularity score of the community detection algorithm. (DOCX)

S1 File The python code used for the analyses. (PY)
