# Supplementary material for: Combining urban scaling and polycentricity to explain socio-economic status of urban regions
Source: PLoS One. 2019 Jun 14;14(6):e0218022. doi: 10.1371/journal.pone.0218022 (PMC6568397; doi:10.1371/journal.pone.0218022)
Supplement: S1 Fig — (DOCX) [file pone.0218022.s001.docx]

**
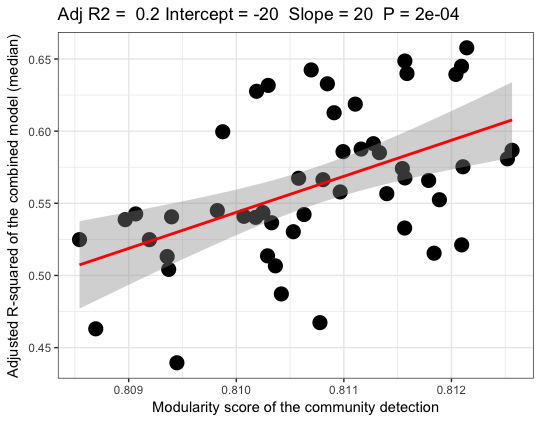

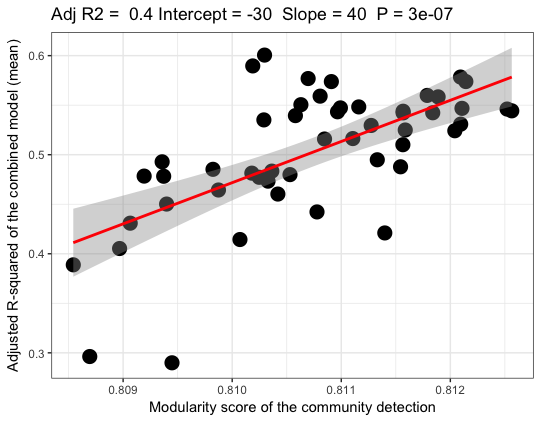
**

S1 Fig: The relationship between the adjusted R-squared of the combined model and modularity score of the community detection algorithm in 50 runs of the community detection algorithm: (Left) Combined model using the mean income as the response variable. (Right) Combined model using the median income as the response variable
